# Optimizing anti-PI3Kδ and anti-LAG-3 immunotherapy dosing regimens in a mouse model of triple-negative breast cancer improves outcome by removing treatment-related adverse events

**DOI:** 10.1136/jitc-2025-012157

**Published:** 2026-02-02

**Authors:** Sarah Nicol Lauder, Ana Pires, Michelle Somerville, Lorenzo Capitani, Kathryn Smart, James Geary, Emily M Mills, Bart Vanhaesebroeck, Andrew Godkin, Awen Gallimore

**Affiliations:** 1 Division of Infection and Immunity, Cardiff University School of Medicine, Cardiff, UK; 2 UCL Cancer Institute, University College London, London, UK

**Keywords:** Immunotherapy, Immune related adverse event - irAE, Breast Cancer, Immune Checkpoint Inhibitor

## Abstract

**Background:**

Current immunotherapy regimens most often fail due to an insufficient T cell response and/or immune-related adverse events (irAEs) which lead to treatment discontinuation. Additionally, many cancers likely require combination immunotherapies which may further increase irAE. This is exemplified in our preclinical models of dual targeting of regulatory T cells with a phosphoinositide 3-kinase δ (PI3Kδ) inhibitor and antibodies to LAG-3. Indeed, while this approach in preclinical models of triple-negative breast cancer shows excellent tumor control, treatment is poorly tolerated and results in significant toxicity. Given the emerging relevance of these targets in human breast cancer, we explored strategies to sustain tumor immunity while mitigating toxicity using these therapeutic modalities.

**Methods:**

Different approaches to combination immunotherapies employing a PI3Kδ inhibitor (PI-3065) with LAG-3 targeting treatments were tested in a mouse model of triple-negative breast cancer to optimize tumor control while limiting irAE.

**Results:**

Systemic targeting of the LAG-3 ligand FGL1 did not provide additional anticancer benefit but markedly worsened irAE. Localized delivery of anti-LAG-3 antibodies to the tumor microenvironment promoted tumor control while reducing the overall number of animals experiencing severe irAE compared with those receiving systemic LAG-3 blockade. However, intermittent dosing of the PI3Kδ inhibitor in combination with anti-LAG-3 treatment prevented the initial development of irAE and enabled excellent tumor control without systemic adverse effects.

**Conclusions:**

Our data demonstrated that refining immunotherapy delivery approaches can improve tolerability that ultimately transforms treatment success.

**WHAT IS ALREADY KNOWN ON THIS TOPIC**

- Previous studies have demonstrated that PI-3065, a small molecule inhibitor of the PI3Kδ signaling pathway, in combination with anti-LAG-3 antibody therapy can promote robust tumor control in preclinical models. This combination approach however has been reported to drive the development of immune-related adverse events (irAEs).

**WHAT THIS STUDY ADDS**

- This study in a preclinical model of triple-negative breast cancer (TNBC), demonstrated that intermittent dosing of PI-3065, combined with systemic inhibition of LAG-3 signaling enables robust tumor regression and prevents the development of treatment-associated irAE. Similar results were obtained by continuous dosing of PI-3065 combined with tumor-localized LAG-3 blockade. In contrast, disruption of LAG-3 signaling via targeting of its ligand, FGL1, failed to control tumor burden and promoted the development of severe irAE.

**HOW THIS STUDY MIGHT AFFECT RESEARCH, PRACTICE OR POLICY**

- This study demonstrates that tumor immunity and the onset of irAEs can be uncoupled by simply altering the dose and/or route of therapy administration. Adoption of this principle, in further translational and clinical studies, could offer novel and better-tolerated therapeutic strategies for patients with cancer.

## Introduction

 Since the first immune checkpoint inhibitor (ICI) was licensed in 2011, these novel immunotherapies have broadened treatment options for cancer with a proportion of patients achieving “miraculous” cures of cancers which previously had been fatal. ICIs are most often antibodies (abs) that target immune checkpoint receptors on T cells, such as programmed cell death protein-1 (PD-1) (pembrolizumab, nivolumab) or cytotoxic T-lymphocytes-associated protein 4 (ipilimumab); they are now widely used in 40% of selected solid cancers, with an overall success rate of 10% in treated patients.^1^ While enabling activation of robust antitumor T cell responses, ICI failure is due to either the development of resistance or due to the side effects of the treatment becoming intolerable, and treatment being aborted.^2 3^ Indeed, ICI therapy can induce a range of immune-related adverse events (irAEs) ranging from mild/transient through to severe/life-threatening.^4 5^ Overcoming irAE is one of the key challenges of ICI therapies.

Regulatory T cells (Tregs) are protumoral due to their suppression of antitumor immune responses and expansion within the tumor microenvironment (TME).^6^ Depletion of Tregs can promote significant tumor clearance in mouse models,^7 8^ however it also results in the development of autoimmunity. Tregs, unlike conventional T cells, are more sensitive to phosphoinositide 3-kinase δ (PI3Kδ) inhibition due to a greater reliance on PI3Kδ signaling for activation and survival pathways,^9^,^11^ making PI3Kδ inhibitors an attractive Treg-targeted therapy. Several studies in preclinical models have demonstrated that PI3Kδ inhibitors significantly reduce or even eradicate tumor burden due to alleviation of Treg immunosuppression.^12^,^14^ The first-approved PI3Kδ inhibitor, idelalisib, was licensed for the treatment of B cell malignancies in 2014, however, its success has been limited due to serious irAE.^15^ Furthermore a clinical trial employing idelalisib for pancreatic ductal adenocarcinoma terminated early due to off-target toxicity.^16^ Nonetheless, the development of second-generation PI3Kδ inhibitors with improved tolerability shows promise. Roginolisib (IOA-244) has encouraging tolerability and efficacy in phase I trials in solid cancers (NCT04328844)^17 18^ with phase II monotherapy and combination therapy commencing recruitment in early 2025 (NCT06879717, NCT06717126, NCT06644183).

Mice rarely exhibit any irAE following ICI, limiting the ability to model irAE development during immunotherapy. Experimental modeling of the grade 3/4 irAE observed in ICI-treated patients typically requires administration of the ICI alongside systemic Treg depletion, or the use of genetically modified strains predisposed to autoimmunity.^19^,^21^ However, the use of PI3Kδ inhibitors in mice recapitulates the irAE observed in patients, with these spontaneously arising on continuous treatment and resolving on cessation of therapy.^13 22^ This accurate reflection of irAE enables elucidation of the mechanisms driving irAE development following PI3Kδ inhibition, either as monotherapy or in combination with novel ICI.

Since reaching the clinic, anti-LAG-3 ab therapy is yet to make a dramatic difference as a monotherapy. Preclinical data from our laboratory suggest that anti-LAG-3 ab treatment in combination with other immunomodulatory therapies can elicit potent tumor control.^13^ These observations have been mirrored in human studies whereby relatlimab, a LAG-3 blocking monoclonal ab, has been successfully combined with nivolumab in advanced melanoma^23^ with the Medicines and Healthcare products Regulatory Agency (MHRA) approving Opdualag (ie, the nivolumab-relatlimab combination) in December 2023 for this patient population.

Despite demonstrating a significant reduction in tumor burden, treatment-associated irAE, particularly dermatological manifestations, is frequently reported with anti-LAG-3/anti-PD1, combination therapy (reviewed by Mullick and Nambudiri^24^), demonstrating the need to continue to develop better tolerated treatment strategies. Conflicting data are emerging on the expression of the LAG-3 ligand, FGL1; predominantly focusing on its role in cancer-type-dependent prognosis and its cellular location of expression. However, in breast cancer, FGL1 is upregulated in comparison to adjacent normal tissues.^25^ Furthermore, elevated FGL1 plasma levels have been associated with poor outcomes in patients treated with PD1/programmed death-ligand 1 (PDL1) therapies.^25^ The progression of immunotherapy has been slower in breast cancer due to its lower immunogenicity compared with other solid tumors such as melanoma. However, PD1 targeting approaches have shown significant clinical benefit in patients who exhibit high expression of PDL1 in the TME,^26^ leading to Food and Drug Administration (FDA) and National Institute for Health and Care Excellence (NICE) approval of the use of pembrolizumab in triple-negative breast cancer (TNBC) in 2021–2022. Early phase clinical trials in TNBC are underway with anti-LAG-3 ab combined with other ICI (NCT03849469, NCT03219268), with the Novartis-funded study reporting curative responses in a patient (NCT02460224) receiving dual PD1 and LAG-3 blockade.^27^ These preliminary findings demonstrate that for TNBC, which has trailed behind other tumor types in the development of immunotherapeutic approaches, the use of combination LAG-3 blockade could significantly improve treatment options.

Here, we extended our previous studies of combination PI3Kδ inhibition and LAG-3 blockade in the 4T1 model of TNBC to uncouple the development of tumor immunity with that of skin-associated irAE. Three different strategies were tested. Firstly, we sought to disrupt the LAG-3 signaling axis by targeting its ligand FGL1, which is expressed at low levels within the skin. second, LAG-3-specific abs were delivered locally to the TME and finally, the PI3Kδ inhibitor was delivered intermittently with systemic LAG-3 blockade. Our findings demonstrate that with the right combination regime, immunotherapies can drive tumor immunity in the complete absence of adverse events.

## Materials and methods

### Mice and cell lines

Female, 8–10 weeks old, BALB/c mice were purchased from Charles River and housed in filter-top cages in specific pathogen-free conditions, with standard chow and water provided ad libitum. Experiments were conducted in accordance with Animal Research Reporting of In Vivo Experiments (ARRIVE) Guidelines V.2.0 and UK Home Office guidelines and were approved by Cardiff University Animal Welfare and Ethical Review Board (AWERB). The 4T1 tumor cell line was obtained from American Type Culture Collection (CRL-2539) while AT-3 cells were obtained from Professor Clare Isacke, ICR. Both were maintained in culture medium (RPMI 1640, 10% FCS, 2 mM L-glutamine, 1 mM sodium pyruvate, and 50 mg/mL penicillin-streptomycin). 1×10^5^ 4T1 cells and 1×10^6^ AT-3 cells were subcutaneously injected into the mammary fat pad. Tumors were measured using digital calipers from day 7 up to three times per week until the end of the experiment. The following calculation was used to determine tumor volume: (Length×Width × Short)×(3.14/6), (where short equals the lower of the length and width measurements and provides an estimate of height).

### In vivo drug treatment

PI-3065 (Advanced ChemBlocks) was administered by oral gavage at a dose of 75 mg/kg, with vehicle-treated mice given an equivalent volume of carrier solution as described previously.^12^ For continuous PI-3065 treatment studies, mice were dosed daily from day −1 prior to tumor inoculation until the termination of the experiment. For intermittent dosing studies, PI-3065 was administered on a 4 days on–3 days off treatment regimen for the duration of the experiment. For combination therapy studies, mice were intraperitoneally administered 250 µg of anti-LAG-3 ab (clone C9B7W, BioXcell) three times per week or 100 µg of anti-FGL1 ab (clone 177R4) two times per week from day 10 onwards. For CD8^+^ depletion studies, mice were administered 200 µg of a CD8-depleting ab (clone YTS169.4, BioXcell), at days 15, 18 and 24 after tumor cell injection. For metastatic control studies mice were administered a combination of 200 µg of a CD8-depleting ab (clone YTS169.4, BioXcell), 200 µg of a CD4-depleting ab (clone GK1.5, BioXcell) and 200 µg of an interferon-gamma (IFNγ) depleting ab (XMG1.2, BioXcell) given intraperitoneally twice a week for 8 weeks at 10 weeks post-tumor rechallenge in long-term controllers. Six to eight mice were included in each treatment arm and experiments were repeated a minimum of twice. Experimental group sizes were calculated from previous experimental data, and^13^ all experimental data generated were reported. Mice were randomly assigned to a treatment group, but researchers were not blinded to treatment.

### Human breast cancer and adjacent tissue samples

Biosamples were obtained from the Wales Cancer Bank (DOI: http://doi.org/10.5334/ojb.46) which is funded by Health and Care Research Wales. Other investigators may have received specimens from the same subjects. Personal data had been processed so that reidentification of the data subject is no longer possible, either directly from the data or from additional information.

### Histology and immunohistochemistry

Tumors and skin samples were excised and fixed in 10% neutral-buffered formalin saline. Fixed tissues were embedded into paraffin and 5 µm sections were cut. For histopathology studies of the skin, sections were stained with H&E and mounted in DPX mountant (Sigma). For fluorescent immunohistochemistry, sections were stained for either the LAG-3 ligands or immune cell infiltrate using an in-house method using the Leica Bond automated staining system. Antigen retrieval was performed using ER2 buffer (Leica Biosystems) prior to endogenous peroxidase activity quenching with 1% H_2_O_2_. Non-specific ab binding was blocked with 10% goat serum. Tumor and skin sections were incubated with the primary abs to MHCII (anti-mouse–M5/114, Bio-Techne, anti-human–LGII-612.14, Cell Signaling Technology), Gal3 (anti-mouse/human–M3/38, BioLegend), FGL1 (anti-mouse–177R4, BioXcell, anti-human–E7C1Q, Cell Signaling Technology), LAG-3 (anti-mouse–ab209238, Abcam, anti-human–BLR028F, Fortis Life Sciences), CD8 (anti-mouse–4SM15, Invitrogen, anti-human–C8/144B, BioLegend), CD4 (anti-human–A19018, ABClonal), F4/80 (anti-mouse–D2S9R, Cell Signaling) or FoxP3 (anti-mouse–D6O8R, Cell Signaling Technology, anti-human–236A/E7, eBioscience) for 50 min at room temperature. Sections were washed and incubated with either anti-Rat ImmPRESS HRP polymer (Vector Labs) or anti-Rabbit VisUCyte detection reagent (Bio-Techne). Slides were incubated briefly with either 488 nm or 594 nm Tyramide conjugates (Biotium) before counterstaining with Hoechst 33342 (Sigma). Slides were washed and mounted in VectaShield (Vector Labs). Sections were imaged at 20× magnification using a Zeiss Axioscan.Z1 slide scanner (Plan-Apochromat 20×/0.8NA). The number of LAG-3 ligand-positive cells and the proximity of CD8^+^ or FoxP3^+^ cells to LAG-3 ligands was quantified using positive cell detection in QuPath as previously described.^28^

### Histopathological scoring

Whole skin sections stained with H&E were scored using an adapted skin scoring system.^29 30^ The following parameters were scored blinded: epidermal thickening (0; normal, 1; minor areas of increased thickening; 2; increased thickening; 3; significantly thickened); epidermal roughening/damage (0; no evidence of damage/roughening, 1; evidence of damage/roughening), epidermal infiltrate (0; normal infiltrate, 1; ≤10% increased infiltrate, 2; ≤25% increased infiltrate, 3; ≤49% increased infiltrate, 4; ≥50% increased infiltrate), dermal infiltrate (0; normal infiltrate, 1; ≤10% increased infiltrate, 2; ≤25% increased infiltrate, 3; ≤49% increased infiltrate, 4; ≥50% increased infiltrate), subcutaneous infiltrate (0; normal infiltrate, 1; ≤10% increased infiltrate, 2; ≤25% increased infiltrate, 3; ≤49% increased infiltrate, 4; ≥50% increased infiltrate). A combined histological score was determined for each sample.

### Survival analysis of TCGA breast adenocarcinoma dataset

RNAseq data in raw counts format and sample meta data were downloaded from The Cancer Genome Atlas (TCGA) using the TCGAbiolinks package in R. Information necessary for tumor subtype stratification (ER status, PR status and HER2 status) was downloaded from the GDC portal. Count normalization was performed using the “limma” package in R^31^ using trimmed of M-mean value normalization after which Principal Component Analysis (PCA) plot of normalized gene counts was generated using the prcomp function, comparing breast cancer to healthy tissue (online supplemental figure 1A). Normalized gene counts of LAG-3 were extracted using the Ensembl gene ID of LAG-3 (ENSG00000089692) and plotted as violin plots comparing healthy tissue to cancer (online supplemental figure 1B).

Subsequently, using the immune deconvolution algorithm CIBERSORTx,^32^ immune cell abundance estimates were generated for all tumor samples. The CD8^+^ T cell estimated abundance was extracted, and samples were split into a “CD8^+^ T cell high” and “CD8^+^ T cell low” based on being in the bottom 50% of top 50% of samples. Using clinical meta data, Kaplan-Meier survival analysis (R packages: “survminer” and “survival”) was performed, using “CD8^+^ T cell group” as a categorical grouping variable. Samples belonging to each “CD8^+^ T cell group” were then split into “LAG-3 high” or “LAG-3 low” groups based on falling in the top 25% or bottom 75% of LAG-3 normalized gene counts respectively. Kaplan-Meier survival analysis was then performed on each “CD8^+^ T cell group”, using “LAG-3 group” as the categorical grouping variable. In each survival analysis, p values were calculated using log-rank testing.

Code is available at https://github.com/LCapitani/TCGA_BRCA_analysis_LAG3

### Statistics and software

Statistical analysis was performed using GraphPad Prism V.10. In all figures, the statistical differences between groups were assessed for normality to determine if a parametric or non-parametric statistical test should be employed. The statistical tests used for each dataset are detailed in the individual figure legends. Unless stated otherwise, data are displayed as the mean±SEM. Statistical significance is denoted as follows: *p<0.05; **p<0.01; ***p<0.001. All schematic images were created in BioRender (https://BioRender.com).

## Results

### LAG-3 is expressed in human breast cancer

**Figure 1:**
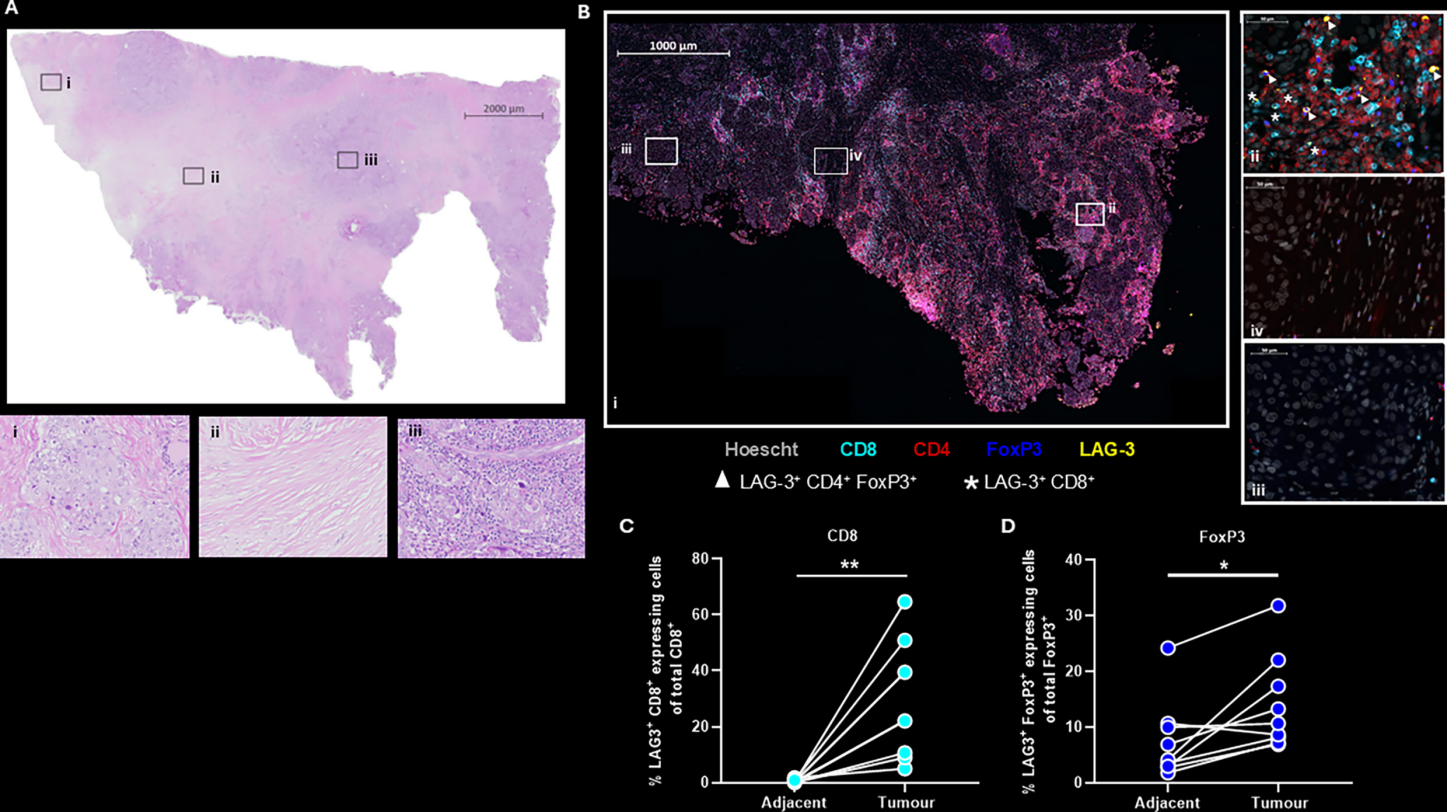
LAG-3 is a relevant marker in human breast cancer. (**A**) H&E stained whole section of a human TNBC paraffin-embedded specimen. (i) Immune cell low area, (ii) stroma rich area, (iii) immune cell high area. (**B**) Fluorescent antigen specific multiplex staining of the TNBC paraffin-embedded specimen depicted in (**A**). (i) whole tissue image, (ii) immune cell rich area, (iii) immune cell low area, and (iv) stroma rich area. Arrows indicate LAG-3^+^ CD4^+^ FoxP3^+^ T cells, asterisks indicate LAG-3^+^ CD8^+^ T cells. (**C**) Comparison of the percentage of CD8^+^ expressing LAG-3^+^ cells from nine matched TNBC and adjacent breast paraffin-embedded specimens. (**D**) Comparison of the percentage of FoxP3^+^ expressing LAG-3^+^ cells from nine matched TNBC and adjacent breast paraffin-embedded specimens (see also online supplemental figure 2). Statistical significance was determined by a Mann-Whitney test (**C and D**) (*p≤0.05, **p≤0.01).

We have previously demonstrated that therapy with PI-3065, a small molecule inhibitor of PI3Kδ enabled robust tumor control in the 4T1 mouse model of TNBC.^13^ Furthermore, when we combined PI-3065 treatment with anti-LAG-3 ab we enabled significantly greater tumor control, with around 50% of dual-treated animals eradicating tumor burden long-term. To determine the potential relevance of LAG-3 as a target in human breast cancer, we interrogated the TCGA database for LAG-3 gene expression in breast cancer (online supplemental figure 1A). In comparison to normal tissue, LAG-3 gene expression was significantly greater in breast cancer (online supplemental figure 1B). Stratification of patients based on high or low tumoral CD8^+^ T cell infiltrate (online supplemental figure 1C) and expression levels of LAG-3 within the TME, revealed better survival outcomes in patients with a high CD8 T cell infiltrate (online supplemental figure 1C), particularly those with the highest LAG-3 expression levels (online supplemental figure 1, compare D to E). Based on this, we surmise that the presence of CD8^+^ T cells and LAG-3 indicates the existence of tumor immunogenicity with subsequent development of counteracting immunosuppressive mechanisms. To examine the expression of LAG-3 at the protein level we used multiplexed ab staining on TNBC tissue samples and matched adjacent breast tissue (figure 1 and online supplemental figure 2). H&E staining revealed the gross pathology of the tissue specimen, showing areas of high and low immune cell infiltrate alongside stroma-rich areas (figure 1A (i) immune cell low area, (ii) stroma-rich area, and (iii) immune cell high area). Multiplexed immunohistochemistry revealed that both CD8^+^ T cells and FoxP3^+^ Treg cells expressing LAG-3 were readily found within the immune-rich areas of the TME (figure 1B). Quantification of nine matched breast cancer and adjacent tissue samples revealed a range of LAG-3 expression on CD8^+^ and FoxP3^+^ T cells between different patients. However, the percentage of LAG-3^+^ CD8^+^ or FoxP3^+^ cells was significantly greater in tumor tissue than in the matched adjacent breast tissue samples (figure 1C,D), demonstrating that LAG-3 expression is highly enriched on T cells in the TME. As observed previously in mice,^13^ LAG-3 expression levels were greatest in CD8^+^ T cells, likely reflecting their activation status in the TME. The enrichment of LAG-3^+^FoxP3^+^ Tregs in tumors also reflects our findings in mice and may represent a population of Tregs with superior suppressive ability as previously described.^33^ Collectively, these data support the notion that LAG-3 is a relevant target for human breast cancer.

### Combined PI3Kδ and LAG-3 blockade promotes robust tumor control but unleashes systemic irAE

**Figure 2:**
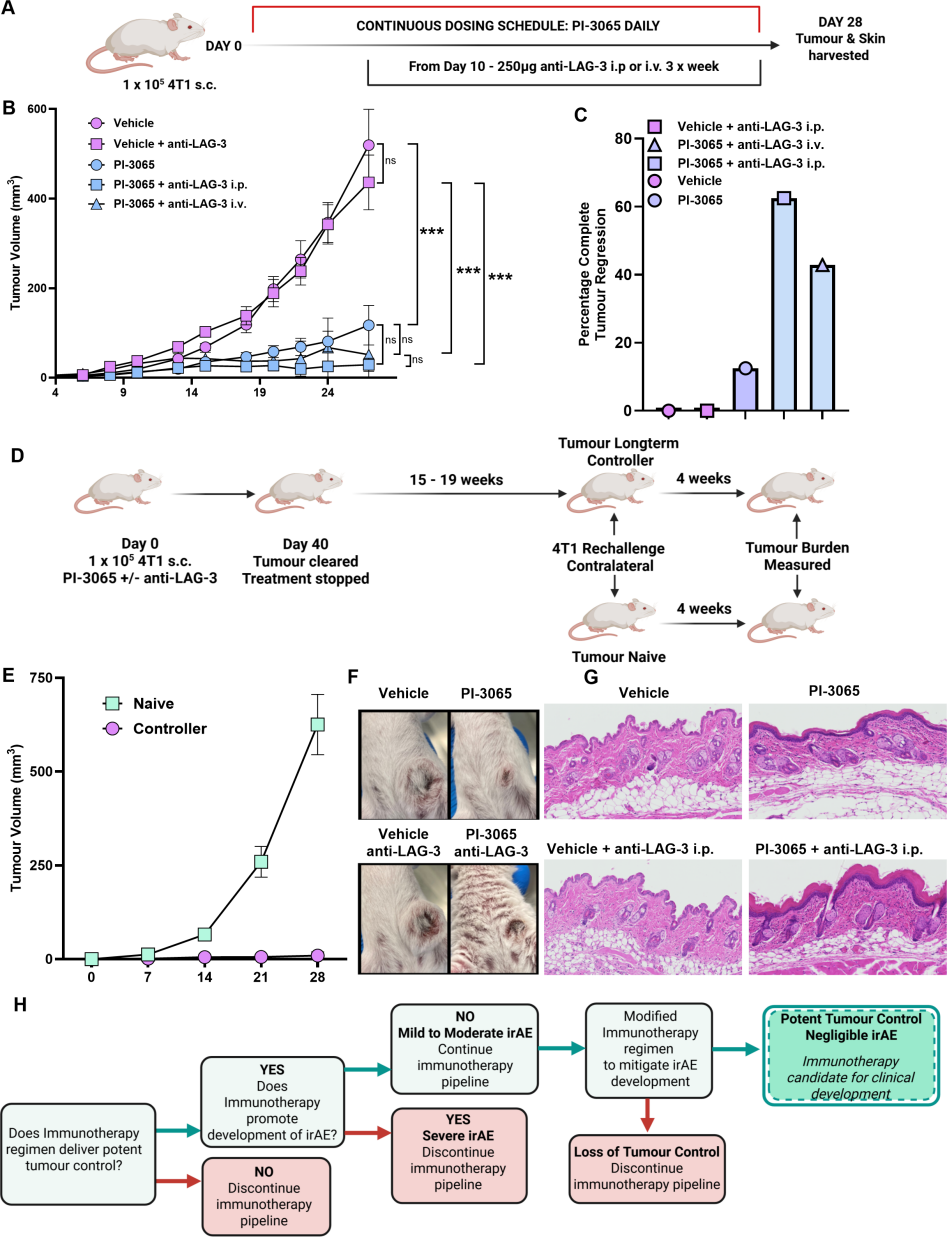
Anti-LAG-3 ab and PI3Kδ combination therapy significantly reduces tumor burden while promoting skin-associated irAE. (**A**) Schematic diagram shows the experimental protocol: Balb/C mice were treated daily for the duration of the study with either 75 mg/kg of PI-3065 or vehicle by oral gavage. 1 day later mice were inoculated with 4T1 tumor cells. Anti-LAG-3 ab was given i.p. or i.v. from day 10, 3 times per week, until tumors were harvested at day 28. (**B**) Tumor growth curves and (**C**) percentage of mice that completely eradicated their tumor burden from each treatment arm (6–8 mice/group). (**D**) Schematic diagram shows the experimental protocol: Balb/C mice were inoculated with 1×10^5^ 4T1 tumor cells. Mice were treated with PI-3065 or PI-3065+anti-LAG-3 ab for 40 days. If total tumor burden was cleared treatment was ceased and mice were monitored for tumor recurrence for 15–19 weeks. These long-term “controllers” were rechallenged alongside tumor-naive mice with 1×10^5^ 4T1 tumor cells and tumor burden measured after 4 weeks. (**E**) Tumor growth curves from controllers and tumor-naive mice following challenge with 1×10^5^ 4T1 tumor cells (n=12/13 mice/group). (**F**) Representative images of skin condition of mice from each treatment group at day 28 post-tumor inoculation (6–8 mice/group). (**G**) Representative skin sections from mice from each treatment group at day 28 post-tumor inoculation, stained with H&E (6–8 mice/group). (**H**) Schematic illustrating the decision pipeline for each proposed therapeutic regimen. All data are displayed as the mean±SEM. Statistical significance was determined by one-way ANOVA (**B**) (*p≤0.05, **p≤0.01, ***p≤0.001).

As previously reported,^13^ using the 4T1 model of TNBC, mice treated with PI-3065 have reduced tumor burden, while mice treated with a combination of PI-3065 and anti-LAG-3 ab can significantly control or even clear established tumors (figure 2A–C). While a greater proportion of mice cleared their tumor in the PI-3065+anti-LAG-3 ab treatment arms, all animals that cleared their tumor (PI-3065 treated or PI-3065+anti-LAG-3 ab treated) developed robust T cell memory responses that protect from subsequent tumor rechallenge several weeks after ceasing treatment (figure 2D,E). In these so-called long-term controllers, tumors appear to be completely eradicated, as removal of the antitumor memory response with depleting anti-CD4, anti-CD8 ab or neutralizing IFNγ did not result in outgrowth of metastatic cells in the lungs, indicating that the initial treatment is curative (online supplemental figure 3A,B). PI-3065 administered alone or in combination with anti-LAG-3 ab did not cause weight loss (online supplemental figure 4A) and histopathological analysis did not reveal any evidence of treatment-induced colitis (data not shown). However, both PI-3065 and combination therapy did elicit skin-associated irAE of varying severity that was macroscopically evident by physical examination of the animals (figure 2F and online supplemental figure 4B). Mice exhibited generalized erythema and piloerection, contributing to overall poor condition of mice given the combination treatment. Histological analysis of skin revealed evidence of epidermal thickening with roughening of the skin surface and significant immune cell infiltration into the epidermal and dermal layers of the skin (figure 2G). Tissue scoring revealed that both single and dual PI-3065+LAG-3 ab treated animals exhibited significantly greater irAE scores than vehicle or anti-LAG-3 ab alone, indicating that PI-3065 treatment is a major driver of irAE. While there was no statistically significant difference in irAE scores between the treated animals, across all experiments performed, the lowest scores were observed in mice treated with PI-3065 alone, while the highest were observed in mice receiving combination therapy (online supplemental table 1). When this experiment was carried out using AT-3 TNBC cells injected into C57BL/6 mice, we found that while PI-3065 treatment significantly improved tumor control and extended the survival time of tumor-bearing mice by approximately 25% in comparison to vehicle-treated mice (online supplemental figure 5A); mice did not develop discernible irAE within the treatment window (online supplemental figure 5B), indicating that, as in patients, the onset of irAEs is most likely influenced by host genetic factors and tumor features.

**Figure 3:**
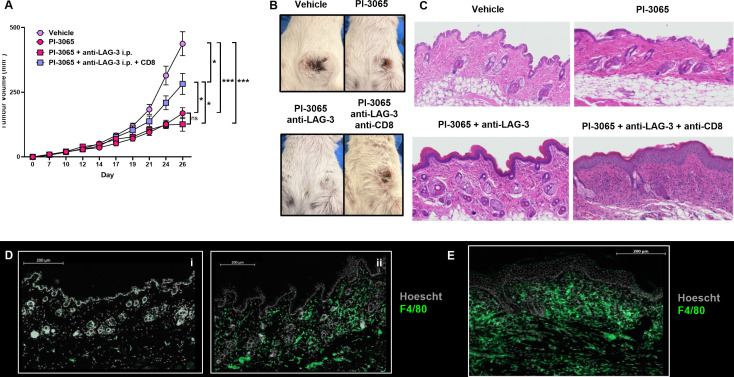
Immune cell profiling of the skin. Balb/C mice were treated daily for the duration of the study with either 75 mg/kg of PI-3065 or vehicle by oral gavage. 1 day later mice were inoculated with 4T1 tumor cells. Anti-LAG-3 ab was given i.p. from day 10, three times per week, until tumors were harvested at day 24. Anti-CD8 depleting abs were administered i.p. at days 15, 18 and 24. (**A**) Tumor growth curves and (**B**) representative images of skin condition of treated mice (n=8 mice/group). (**C**) Representative skin sections from mice from each treatment group at day 24 post-tumor inoculation, stained with H&E (8 mice/group). Fluorescent ab multiplex staining depicting F4/80^+^ cells in the skin of vehicle-treated (i) and PI-3065+anti-LAG-3 ab-treated (ii) mice (**D**). Representative fluorescent ab multiplex staining depicting F4/80^+^ staining in the skin of PI-3065+anti-LAG-3 ab+anti-CD8 treated mice (**E**). All data are displayed as the mean±SEM. Statistical significance was determined by one-way ANOVA (A) (* p≤0.05, *** p≤0.001).

To determine whether the same effector cells were driving both tumor immunity and irAE development in Balb/C mice, we tested whether CD8^+^ T cells, shown in our previous study to be essential for tumor rejection in PI-3065 and PI-3065+anti-LAG-3 ab treated mice^13^ (figure 3A), also mediated irAEs. Following treatment of these mice with depleting CD8-specific abs, we found the converse to be true; depleting CD8^+^ cells exacerbated cutaneous irAE in animals treated with PI-3065+anti-LAG-3 ab combination therapy (figure 3B,C). Immune profiling of the skin from PI-3065+anti-LAG-3 ab treated mice revealed that the cutaneous inflammation observed was associated with an infiltration of F4/80^+^ cells (figure 3D). Moreover, depletion of CD8^+^ cells increased the infiltration of F4/80^+^ cells in skin (figure 3E), implying that CD8^+^ cells may play a role in maintaining immune homeostasis at this site. Collectively these data indicate that different effector cells promote tumor immunity and skin inflammation.

In summary, our data thus far showed that PI-3065+anti-LAG-3 ab combination therapy had the potential to elicit curative tumor control in a significant proportion of treated mice but could also drive pronounced irAE, diminishing its attractiveness as an immunotherapy for patients with cancer. Using the decision pipeline delineated in figure 2H, we sought to test therapeutic regimens, that aimed to promote robust tumor control, while mitigating against the development of irAEs.

### Exploring the LAG-3 ligand FGL1 as an alternative target for immunotherapy

To try to circumvent combination therapy irAE, we explored LAG-3 ligands as alternative targets to disrupt LAG-3 signaling. LAG-3 has five known ligands, namely LSECtin, α-synuclein, Gal-3, MHCII, and FGL1^34^ of which MHCII, Gal-3 and FGL1 have been the most widely studied in tumors.

**Figure 4:**
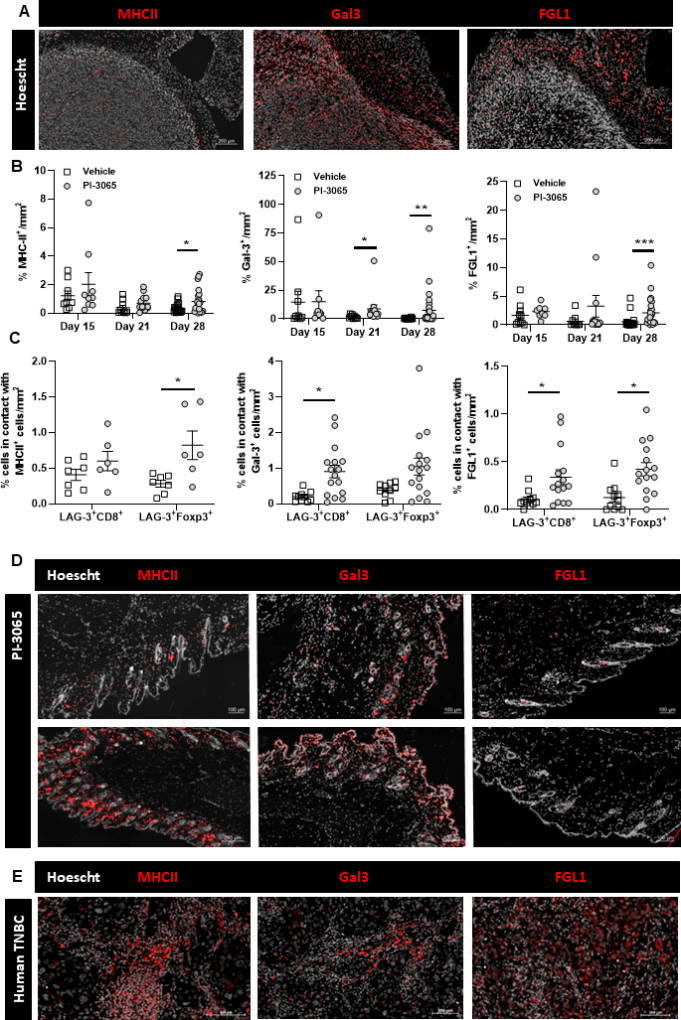
LAG-3 ligand expression in mouse and human tumors. (**A**) Tumor expression of MHCII, Gal3 and FGL1. (**B**) Quantification of MHCII, Gal3 and FGL1 in the tumors at days 15, 21 and 28 post-tumor inoculation. (**C**) Quantification of LAG-3 expressing CD8^+^ T cells and FoxP3^+^ Tregs in direct contact with MHCII, Gal3 and FGL1 expressing cells within the tumor. (**D**) Representative expression of MHCII, Gal3 and FGL1 in the skin of vehicle or PI-3065 treated tumor bearing mice at day 28 postinoculation. (**E**) Representative expression of MHCII, Gal3 and FGL1 in human TNBC tumors. All data is displayed as the mean±SEM. Statistical significance was determined by a Mann-Whitney test (B) or Unpaired T test (C) (* p≤0.05, ** p≤0.01, *** p≤ 0.001).

The expression levels of MHCII, Gal-3 and FGL1 were examined in tumors of untreated and PI-3065-treated mice at various time points post-tumor inoculation. Gal-3 showed the highest expression within the TME, located both deep within and at the tumor periphery (figure 4A). FGL1-positive cells tended to be located closer to the tumor margins, while MHCII expression was expressed at low levels throughout the TME. Quantification of ligand expression levels revealed that PI-3065 treatment promoted the expression of all three ligands, demonstrable at day 28 post-tumor inoculation (figure 4B). To determine if the ligands within the tumor are potentially initiating LAG-3 signaling, the proximity of both LAG-3-expressing CD8^+^ and FoxP3^+^ cells to each ligand was established (figure 4C). FGL1^+^ cells in contact with both LAG-3^+^ CD8^+^ and LAG-3^+^ FoxP3^+^ were significantly greater in PI-3065-treated than in vehicle-treated mice, suggesting that blockade of FGL1 could prevent LAG-3 signaling in PI-3065-treated mice. To help predict whether ligand blockade would cause skin inflammation, we also examined expression of MHCII, Gal-3 and FGL1 in skin samples. Both MHCII and Gal-3 were widely expressed in the skin, whereas FGL1 expression was low (figure 4D). Finally, we assessed human TNBC samples and found that MHCII, Gal-3 and FGL1 were all expressed in the TME (figure 4E). Overall, the high expression and proximity of FGL1-expressing cells to both CD8^+^ and FoxP3^+^ LAG-3^+^ immune cells within the tumor, combined with the low expression of FGL1 within the skin implied that of the three ligands tested, FGL1 was the most promising candidate for blockade in vivo.

### Combination PI-3065 and FGL1 blockade enables tumor control yet induces skin-associated irAE

**Figure 5:**
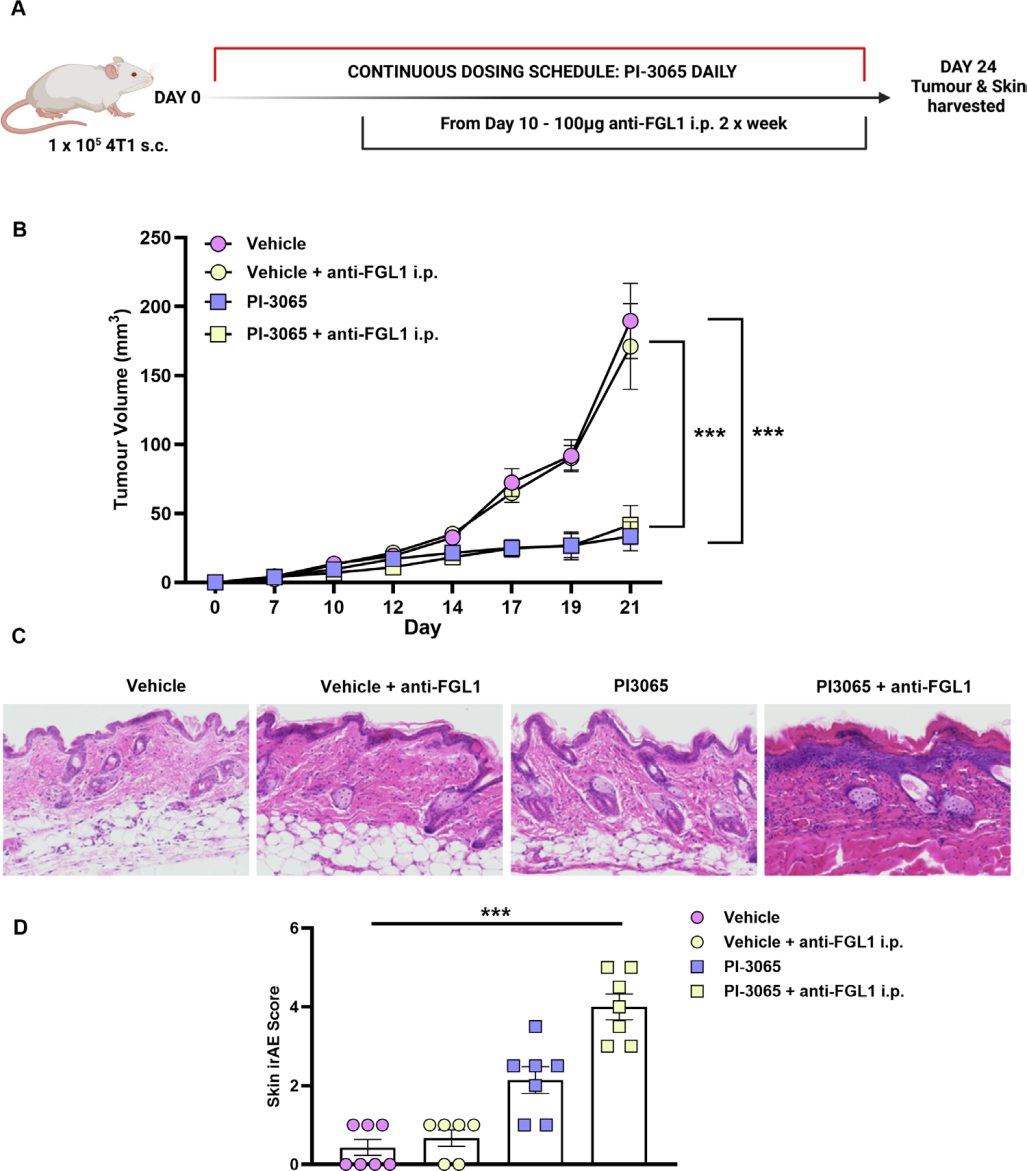
Combination anti-FGL1 antibody therapy promotes tumor control but does not reduce skin-associated irAE. (**A**) Schematic diagram shows the experimental protocol: Balb/C mice were treated daily for the duration of the study with either 75 mg/kg of PI-3065 or vehicle by oral gavage. 1 day later mice were inoculated with 4T1 tumor cells. Anti-FGL1 ab (100 ug) was given i.p. from day 10, two times per week until tumors were harvested at day 21–24. (**B**) Tumor growth curves of mice from each treatment arm (8 mice/group). (**C**) Representative images of skin condition of mice from each treatment group at day 24 post-tumor inoculation (8 mice/group). (**D**) Skin histology scores from mice at day 24 post-tumor inoculation (6–8 mice/group). All data are displayed as the mean±SEM. Statistical significance was determined by one-way ANOVA (***p≤0.001).

To test the efficacy of FGL1 blockade, mice were treated with PI-3065 daily in combination with 100 µg of anti-FGL1 abs administered from day 10 onwards (figure 5A). Anti-FGL1 ab alone did not confer tumor control, and when administered in conjunction with PI-3065, did not promote better tumor control than PI-3065 on its own (figure 5B). Moreover, they failed to elicit the level of tumor control observed in dual PI-3065 and anti-LAG-3 ab treated mice (online supplemental figure 6). However, skin-associated irAE was significantly heightened, with experimental mice on dual treatment terminated between day 21 and 24 due to the severity of the skin inflammation observed (figure 5C,D). Repeated administration of anti-FGL1 ab was poorly tolerated with animals rapidly exhibiting severe somnolence ± seizures following systemic dosing. These unexpected off-target side effects were greatest in vehicle-treated mice that were otherwise fit and well, but tumor burden was significantly greater than in PI-3065-treated mice. These data demonstrate that alternate targeting of the LAG-3 signaling via FGL1 provides inferior control of tumor growth, and moreover, exacerbates irAE induced through PI-3065 alone. Given the poor tumor control with continuous anti-FGL1 ab and PI-3065, we opted to discontinue this therapeutic regimen in accordance with our treatment decision pipeline (figure 2H)

### Tumor-localized LAG-3 blockade mitigates the irAE observed with systemic LAG-3 targeting

**Figure 6:**
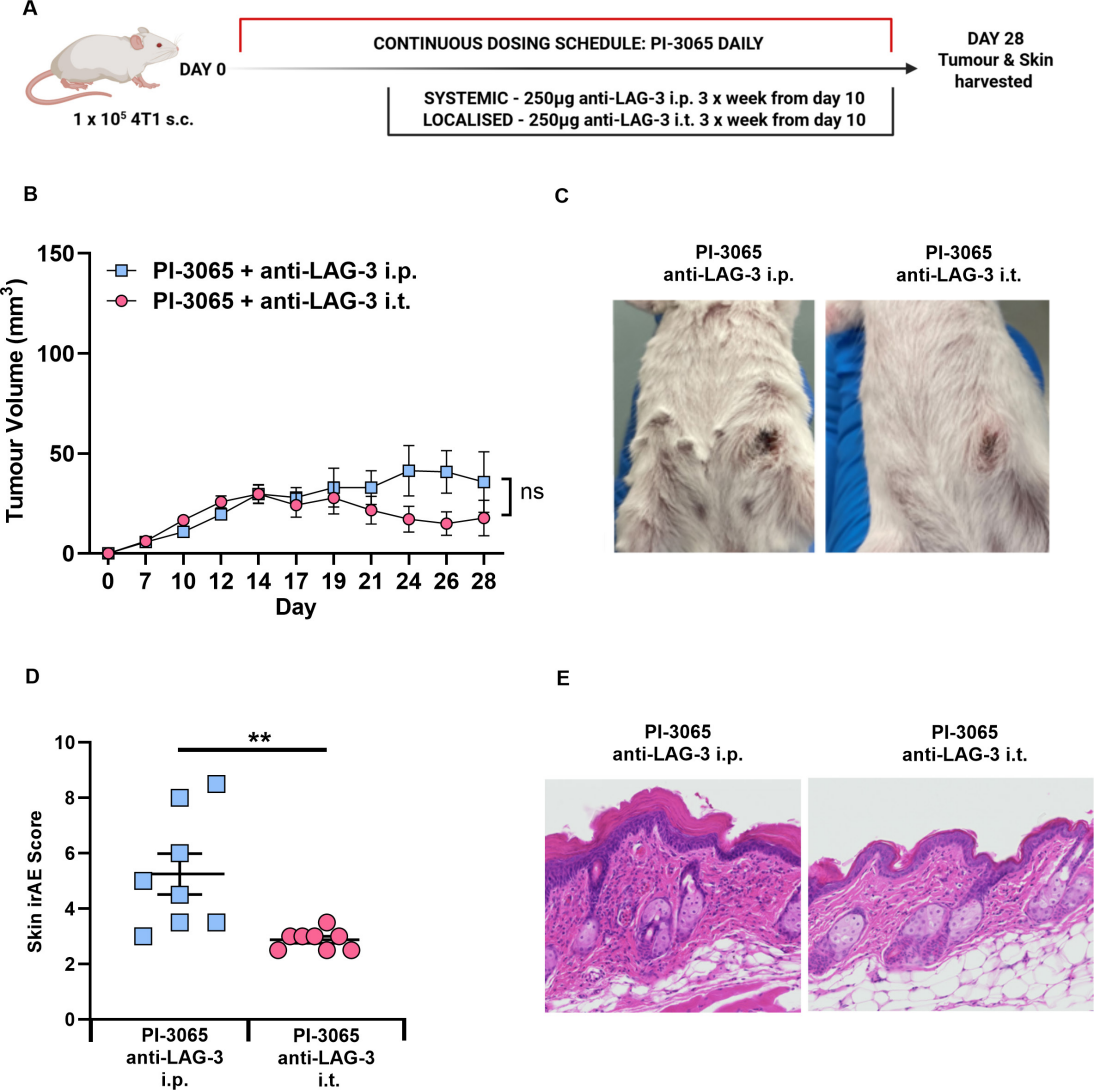
Localized administration of anti-LAG-3 ab confers tumor control but ameliorates irAE. (**A**) Schematic diagram shows the experimental protocol: Balb/C mice were treated daily for the duration of the study with either 75 mg/kg of PI-3065 or vehicle by oral gavage. 1 day later mice were inoculated with 4T1 tumor cells. Anti-LAG-3 ab (250 μg) was administered either i.p. or i.t. from day 10, three times per week, until tumors were harvested at day 28. (**B**) Tumor growth curves of mice treated with PI-3065 with anti-LAG-3 ab administered either systemically (i.p.) or locally (i.t.) (8 mice/group). (**C**) Representative images of skin condition of mice treated with PI-3065+anti-LAG-3 ab i.p. or PI-3065+anti-LAG-3 ab i.t. (**D**) Skin histology scores from mice at day 28 post-tumor inoculation (**E**) Representative skin sections stained with H&E from mice treated with PI-3065+anti-LAG-3 ab i.p. or PI-3065+anti-LAG-3 ab i.t. All data are displayed as the mean±SEM. Statistical significance was determined by two-way ANOVA (**B**) or unpaired t-test (**D**). (*p≤0.05, **p≤0.01, ***p≤0.001).

We next tested whether localized administration of anti-LAG-3 ab avoids the skin-associated irAEs observed, while still promoting tumor clearance. Anti-LAG-3 ab was injected directly into the tumor (figure 6A) in combination with PI-3065 and provided comparable anticancer effects as systemic treatment (figure 6B). Tumors regressed in 70% of the combination-treated mice with localized anti-LAG-3 ab therapy compared with 66% of mice treated with systemic anti-LAG-3 ab. Localized anti-LAG-3 ab alone did not reduce tumor burden in any treatment setting (data not shown). Mice treated with PI-3065 and intratumoral anti-LAG-3 ab exhibited no overt signs of irAE, with no erythema or piloerection present (figure 6C). Analysis of skin samples demonstrated a significant improvement in overall pathology, with a reduction in both thickening of the epidermal layer, and immune cell infiltrate into the epidermis and dermis, that contributed to an overall reduction in the skin irAE score (figure 6D,E).

### Intermittent dosing of PI-3065 offers potent tumor control and negligible irAE

**Figure 7:**
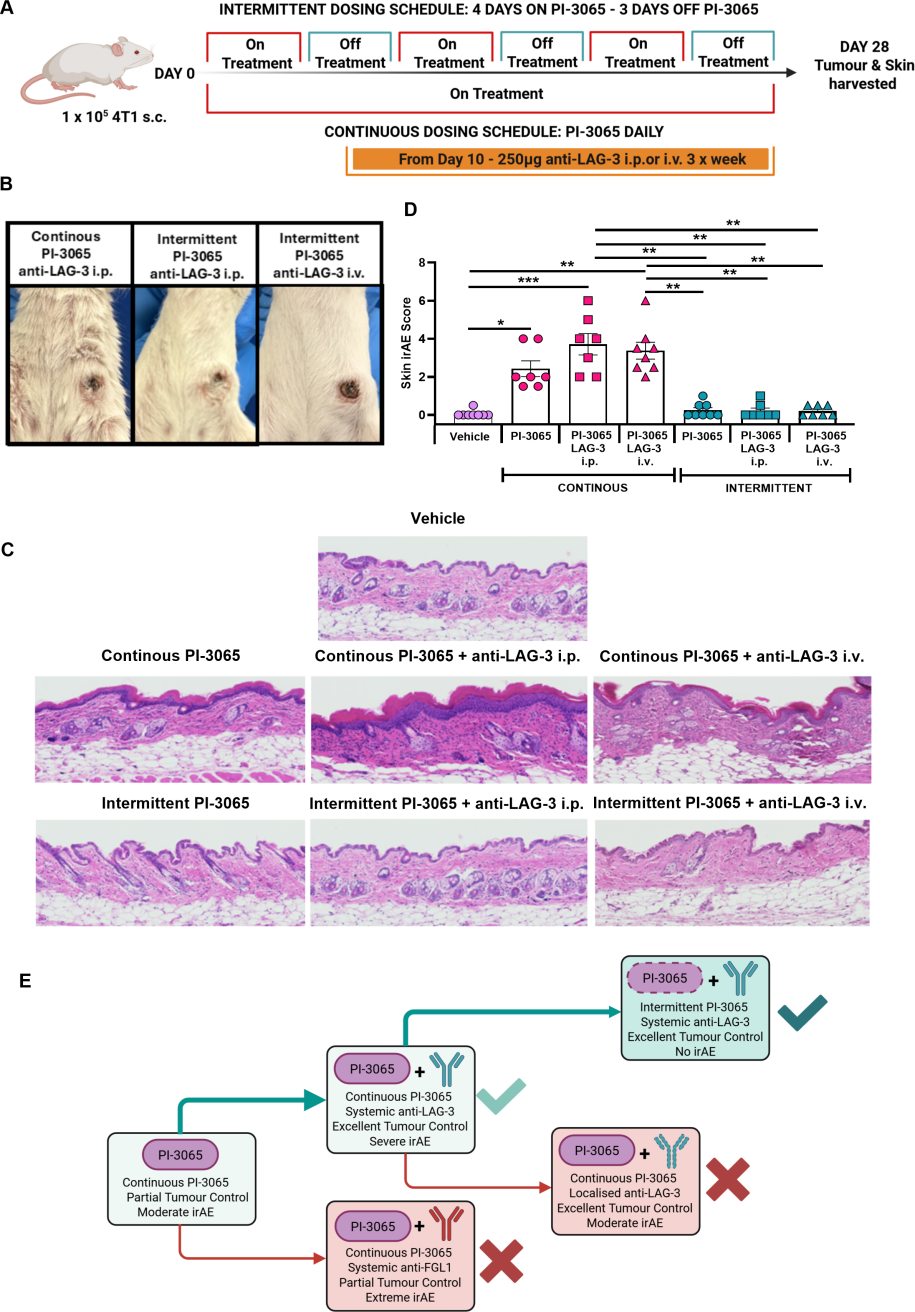
Intermittent PI-3065 therapy combined with anti-LAG-3 ab offers potent tumor control and negligible irAE. (**A**) Schematic diagram shows the experimental protocol: Balb/C mice were either treated daily with 75 mg/kg PI-3065 or treated for 4 consecutive days followed by a 3-day respite period of no treatment, repeated for the duration of the study. Anti-LAG-3 ab (250 mg) was administered i.p. or i.v. from day 10, three times per week until tumors were harvested. (**B**) Representative images of skin condition of mice treated with either continuous PI-3065+anti-LAG-3 or intermittent PI-3065+anti-LAG-3 ab. (**C**) Representative skin sections stained with H&E from mice treated with vehicle, continuous PI-3065, continuous PI-3065+anti-LAG-3 ab, intermittent PI-3065 or intermittent PI-3065+anti-LAG-3 ab. (D) Skin histology scores from the different treatment groups. (E) Schematic illustrating the optimal therapeutic strategy for PI-3065+anti-LAG-3 ab combination therapy. All data displayed as the Mean±SEM. Statistical significance was determined by one-way ANOVA (*p≤0.05, **p≤0.01, ***p≤0.001).

Our studies demonstrate that PI-3065 treatment alone also drives development of skin-associated irAE, exacerbated by systemic but not by localized LAG-3 blockade, indicating that PI-3065 was the primary driving factor behind the inflammation evident in the skin. A previous report in a mouse model of melanoma that exhibited colitis with PI3Kδ inhibition demonstrated that intermittent PI-3065 dosing decreased the gastrointestinal toxicity observed with continuous dosing.^22^ We therefore next explored whether skin-associated irAE could be reduced by intermittent PI-3065 dosing in combination with systemically administered anti-LAG-3 ab. Mice were treated with 75 mg/kg of PI-3065 orally using a “4 days on–3 days off” treatment regime for the duration of the study with the addition of systemic LAG-3 ab, administered from day 10 onwards (figure 7A). Systemic administration of anti-LAG-3 ab together with either continuous or intermittent PI-3065 treatment promoted robust tumor control and clearance. Moreover, mice administered intermittent PI-3065 alone or in combination with anti-LAG-3 ab were unremarkable with no evidence of skin-associated irAE (figure 7B). Histological analysis of skin samples taken from intermittent PI-3065 treated mice alone or with LAG-3 blockade revealed minor areas of epidermal thickening and roughening, with no epidermal or dermal immune cell infiltration, resulting in a significantly reduced skin irAE score compared with mice receiving continuous PI-3065 treatment (figure 7C,D).

## Discussion

The impact of cancer immunotherapy is tempered by poor responses and/or treatment-associated toxicities. In our previous study that sought to interfere with Treg function using the PI3Kδ inhibitor, PI-3065, we found that some Tregs residing in the TME of treated mice upregulated expression of LAG-3, possibly reflecting a potential immune escape mechanism.^13^ Blockade of LAG-3 in combination with PI-3065 treatment offered powerful tumor control, with 50% of animals clearing tumor burden completely. However, PI3Kδ inhibition induces skin-associated irAE with a spectrum of severity with the lowest scores observed in mice treated with PI-3065 alone and the highest in mice receiving both PI-3065 and anti-LAG-3 abs, likely due to a breach in immune homeostasis within the skin. This spectrum of severity in both mono and combination therapy treated animals reflects the irAE observed in patients treated with checkpoint inhibitors; some patients tolerate therapy with minimal side effects, while others develop irAE that can be life-threatening. Understanding the mechanisms of irAE development and predicting these responses in patients is challenging, therefore treatment regimens that offer minimal irAE are highly sought. Here, we report that altering either the dosing strategy or the route of administration of these therapies ameliorates irAE while preserving effective antitumor immune responses.

While the immunotherapy field continues to evolve at pace, reducing off-target effects through the development of tumor-targeted ICI is an important therapeutic strategy (reviewed by Melero *et al*^35^). In our hands, intratumoral delivery of anti-LAG-3 in combination with systemic PI-3065 enabled robust tumor control but reduced the severity of skin irAE to the levels typically observed with PI-3065 treatment alone. While this approach experimentally confirmed that local delivery offers improved tolerability, clinically administering abs directly to the tumor is only suitable as a therapy for accessible tumors that are permissive to direct ab targeting. Improved systemic therapies that directly target the tumor to deliver their anti-LAG-3 payload, such as bispecific abs or oncolytic viruses in combination with systemic PI3Kδ inhibition warrant further study to determine if this approach could be translated to the clinic.

Patients treated with the first-generation PI3Kδ inhibitor idelalisib frequently show moderate-to-severe skin rashes following treatment.^36^ To determine whether the development of irAE and the generation of robust antitumor immunity was mediated by the same effector cells, we depleted CD8^+^ T cells from tumor-bearing mice receiving continuous combination therapy. While CD8^+^ T cells are essential for tumor rejection, their removal significantly exacerbated skin inflammation, demonstrating that the development of a robust antitumor response is not a requisite for the generation of skin-associated irAE. Indeed, the irAE in the skin of treated mice associated with a pronounced infiltration of F4/80^+^ cells that are most likely macrophages. While the mechanisms behind irAE in patients are yet to be fully elucidated, there is evidence that infiltrating T cells and macrophages can promote irAE development through cytokine-dependent pathways.^37^ Furthermore, histological analysis of skin samples taken from ICI-treated patients experiencing cutaneous irAE revealed increased frequencies of M2 macrophages within the skin that promoted tissue dysregulation.^38^ Curiously, the inflammatory response observed in the mice described in our study, was also kept in check by CD8^+^ cells. Studies of mouse models of skin inflammation indicate that classical CD4^+^ Tregs and a small population of CD8^+^ FoxP3^+^ Tregs (CD8 Regs) play a role in maintaining peripheral tolerance at this site.^39^

Given the poor tolerability of idelalisib, there has been significant endeavors to find novel PI3Kδ inhibitors that provide comparable clinical benefit with an improved safety profile (reviewed by Belli *et al*^40^). Next-generation PI3Kδ inhibitors such as zandelisib, parsaclisib and especially roginolisib have been associated with reduced incidence of grade 3/4 irAE during early-stage clinical trials (iOnctura; NCT04328844^41^,^43^). Recent preclinical and clinical studies have demonstrated improved tolerance of PI3Kδ inhibition by intermittent dosing strategies.^22 41^ Using the B16-F10 model of melanoma, Eschweiler and colleagues were able to significantly reduce tumor burden following continuous treatment with PI-3065, yet this was associated with the development of colitis.^22^ They further showed that an intermittent dosing led to tumor control without significant colitis.^22^ Carnavelli *et al* had previously demonstrated in a range of preclinical models, that intermittent dosing of either PI-3065, or the PI3Kα/δ dual inhibitor, AZD8835, enabled potent tumor control with no reported adverse events.^44^ Our data also demonstrate that intermittent dosing significantly improved the skin-associated irAE observed while maintaining robust tumor control. Together these data point towards PI3Kδ inhibition being pivotal in the development of irAE. Previous data have demonstrated that PI3Kδ is essential for Treg survival and function.^45 46^ Employing an intermittent dosing strategy is sufficient to reduce the Treg-mediated suppression within the TME but does not disrupt the delicate balance of tolerance in the periphery, preventing the development of serious irAE. PI3Kδ inhibition results in the development of a range of irAE, dependent on the genetic background of the mouse strain employed.^47 48^ The use of different wild-type strains of mice allows the most common irAE observed in patients to be modeled and the mechanisms behind their development examined.

We also attempted to mitigate against irAE driven by LAG-3 blockade by targeting its FGL1 ligand that is expressed only at very low levels in the skin. FGL1 is a hepatokine expressed in the liver that in the steady state mediates several cellular processes such as proliferation and metabolism of hepatocytes. Following liver injury, FGL1 is considered protective, promoting mitochondrial mitosis and regeneration of liver cells.^49^ Several studies have reported that FGL1 blockade can promote antitumor effects.^25 50^ In our studies we found that alone and in combination with PI-3065, anti-FGL1 ab therapy offered less improvement in tumor control compared with anti-LAG-3 ab. Moreover, systemic anti-FGL1 ab therapy induced swift and severe side effects in animals with high tumor burden. Previous reports in the literature report no adverse effects of anti-FGL1 ab therapy. We suspect that the side effects in our study are driven by the ab targeting tissues with high expression of FGL1. 4T1 tumors are known to rapidly metastasize to a range of tissues, including the liver, which is the primary tissue source of FGL1 under normal physiological conditions. It is possible that high FGL1 produced by hepatocytes, coupled with liver injury induced by metastatic cells within the liver results in the development of acute fulminant hepatic failure when the liver-protective effect of FGL1 is blocked by ab treatment. Indeed, the severe somnolence and seizures observed in the animals would be in keeping with hepatic encephalopathy, a key manifestation of hepatic failure. Uncovering the detailed mechanisms behind these effects is beyond the scope of this study, however, life-threatening hepatic side effects are a known irAE of other immunotherapy modalities.^51 52^ Previous reports have demonstrated that FGL1-LAG-3 signaling is essential to maintain immune tolerance and to prevent autoimmunity.^25^ Indeed, recombinant FGL1 reduced disease severity in a murine collagen-induced arthritis model,^53^ while reduced FGL1 levels in vivo promoted the production of proinflammatory cytokines such as tumor necrosis factor alpha, interleukin (IL)-1β and IL-6, and exacerbated hepatitis development.^54^ FGL1-KO animals are healthy during infancy, however with aging they develop spontaneous dermatitis and plasma dsDNA abs, indicative of the key role of FGL1 in maintaining peripheral tolerance.^25^ While the role of FGL1 in maintaining skin tolerance is yet to be fully elucidated, it is known that FGL1 plays an essential role in the regulation of the hormone hepcidin.^55^ A recent study has demonstrated that hepcidin expression within the skin promoted psoriasis in a transgenic mouse model.^56^ Disruption of FGL1 signaling, either via membrane-bound or soluble hepatocyte-derived FGL1, may promote similar skin-associated inflammation via a FGL1-hepcidin pathway. While exercising caution over the use of anti-FGL1 ab therapy in cancer immunotherapy, we did not test whether local intratumoral treatment with this ab might mediate antitumor effects without toxicity.

Taken together our study demonstrates that intermittent inhibition of PI3Kδ in combination with LAG-3 blockade can promote rejection of primary tumors and long-term control of disease, while mitigating against the development of irAE. The most important finding of our study is that tumor immunity and the onset of irAEs can be uncoupled by simply altering the dose and/or route of therapy administration. Adoption of this principle, in further translational and clinical studies, could offer significant benefits for patients with cancer.

## Supplementary material

10.1136/jitc-2025-012157 online supplemental figure 1

10.1136/jitc-2025-012157 online supplemental figure 2

10.1136/jitc-2025-012157 online supplemental figure 3

10.1136/jitc-2025-012157 online supplemental figure 4

10.1136/jitc-2025-012157 online supplemental figure 5

10.1136/jitc-2025-012157 online supplemental figure 6

10.1136/jitc-2025-012157 online supplemental table 1

10.1136/jitc-2025-012157 online supplemental file 1

## Data Availability

All data relevant to the study are included in the article or uploaded as supplementary information.
