# Supplementary figures and images for: Optimizing anti-PI3Kδ and anti-LAG-3 immunotherapy dosing regimens in a mouse model of triple-negative breast cancer improves outcome by removing treatment-related adverse events
Source: J Immunother Cancer. 2026 Feb 2;14(2):e012157. doi: 10.1136/jitc-2025-012157 (PMC12878477; doi:10.1136/jitc-2025-012157)

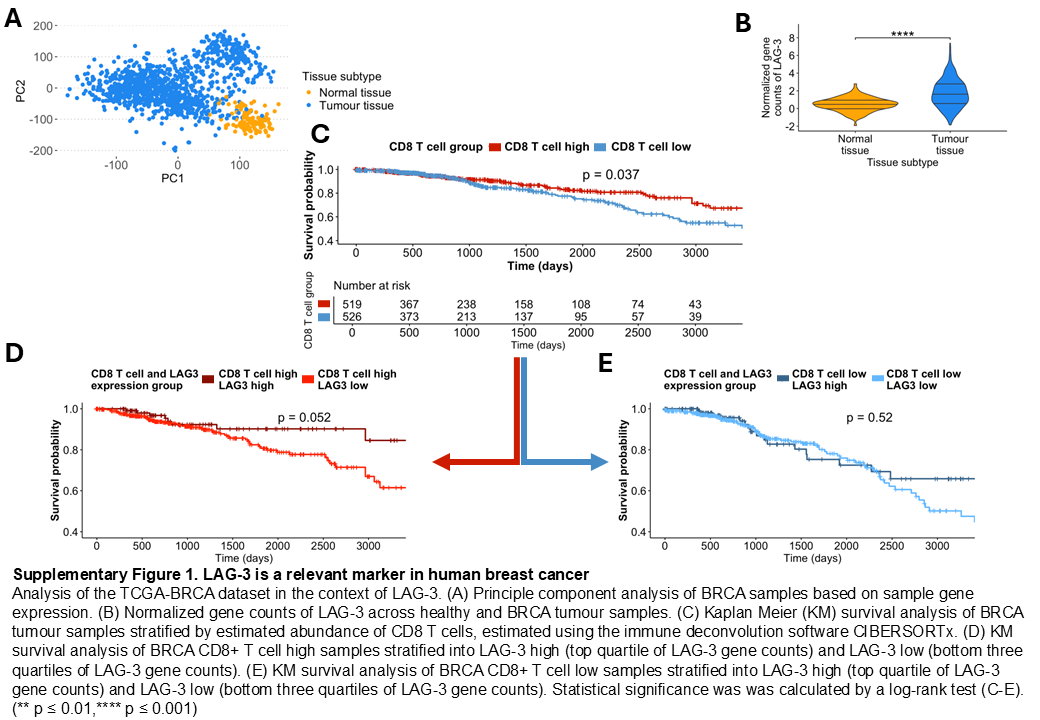

Supplement: online supplemental figure 1 [file jitc-14-2-s001.tif]

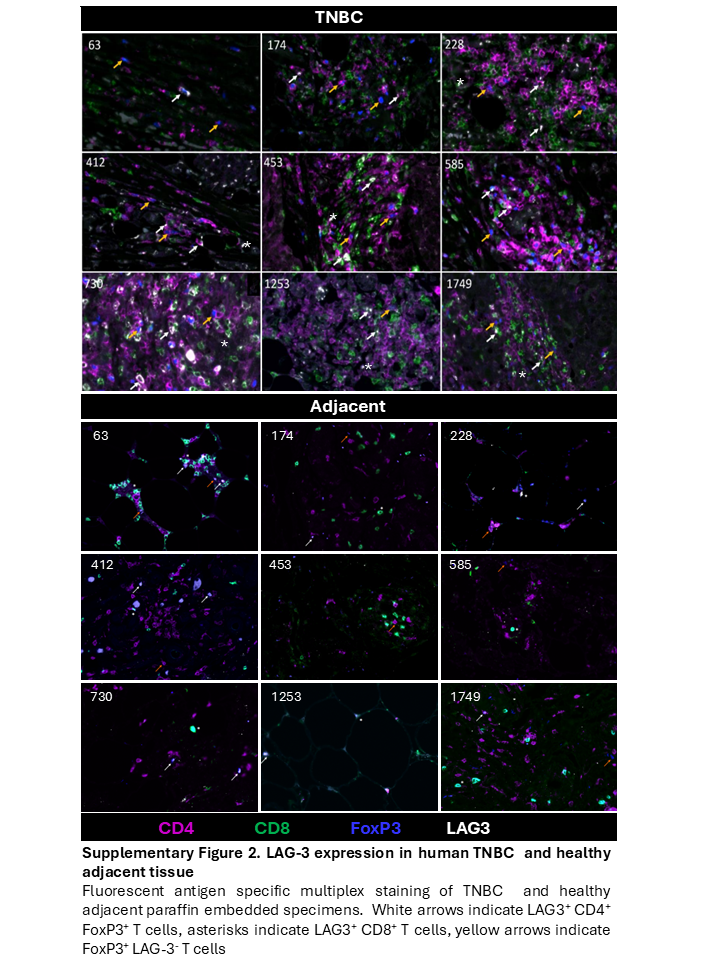

Supplement: online supplemental figure 2 [file jitc-14-2-s002.tif]

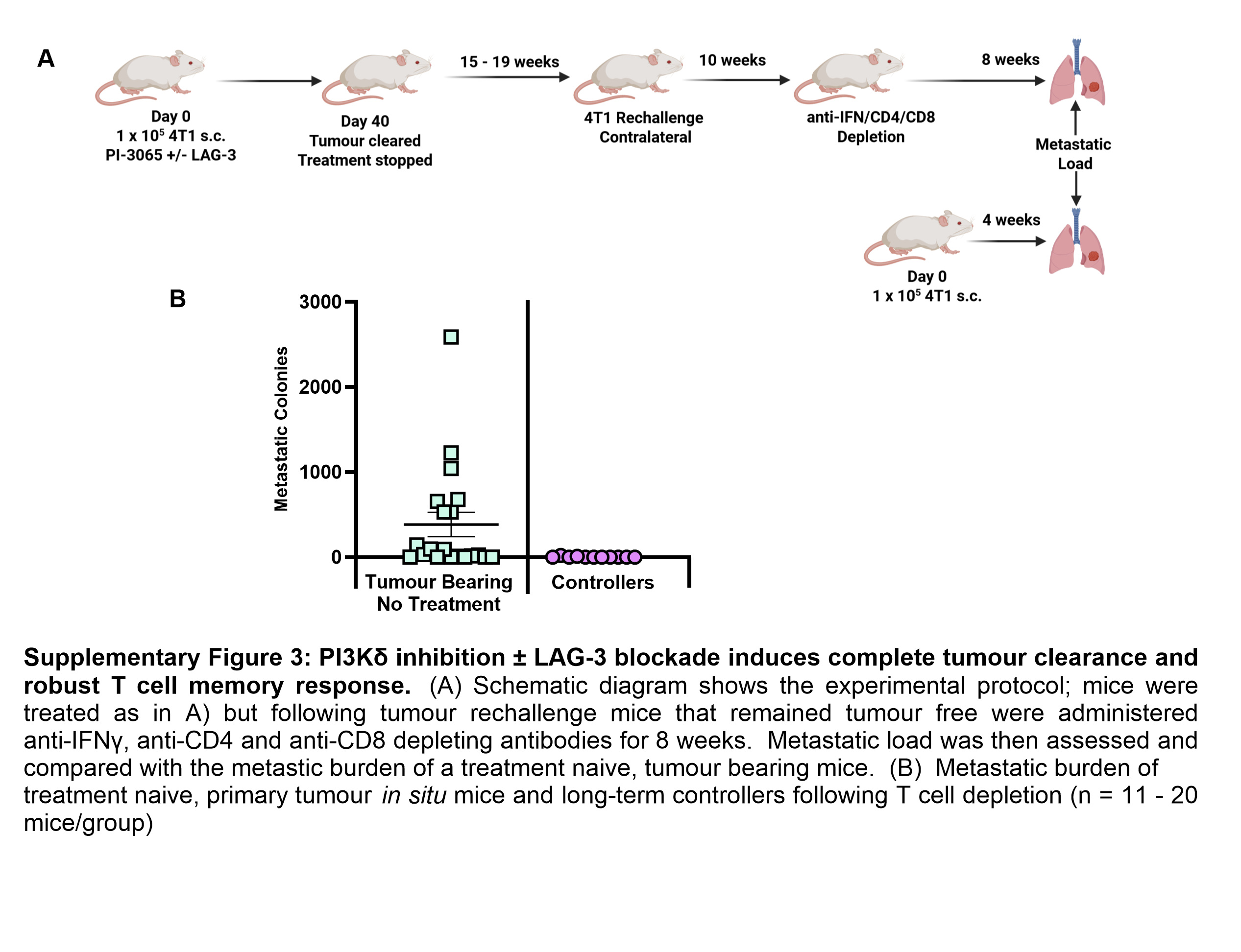

Supplement: online supplemental figure 3 [file jitc-14-2-s003.tif]

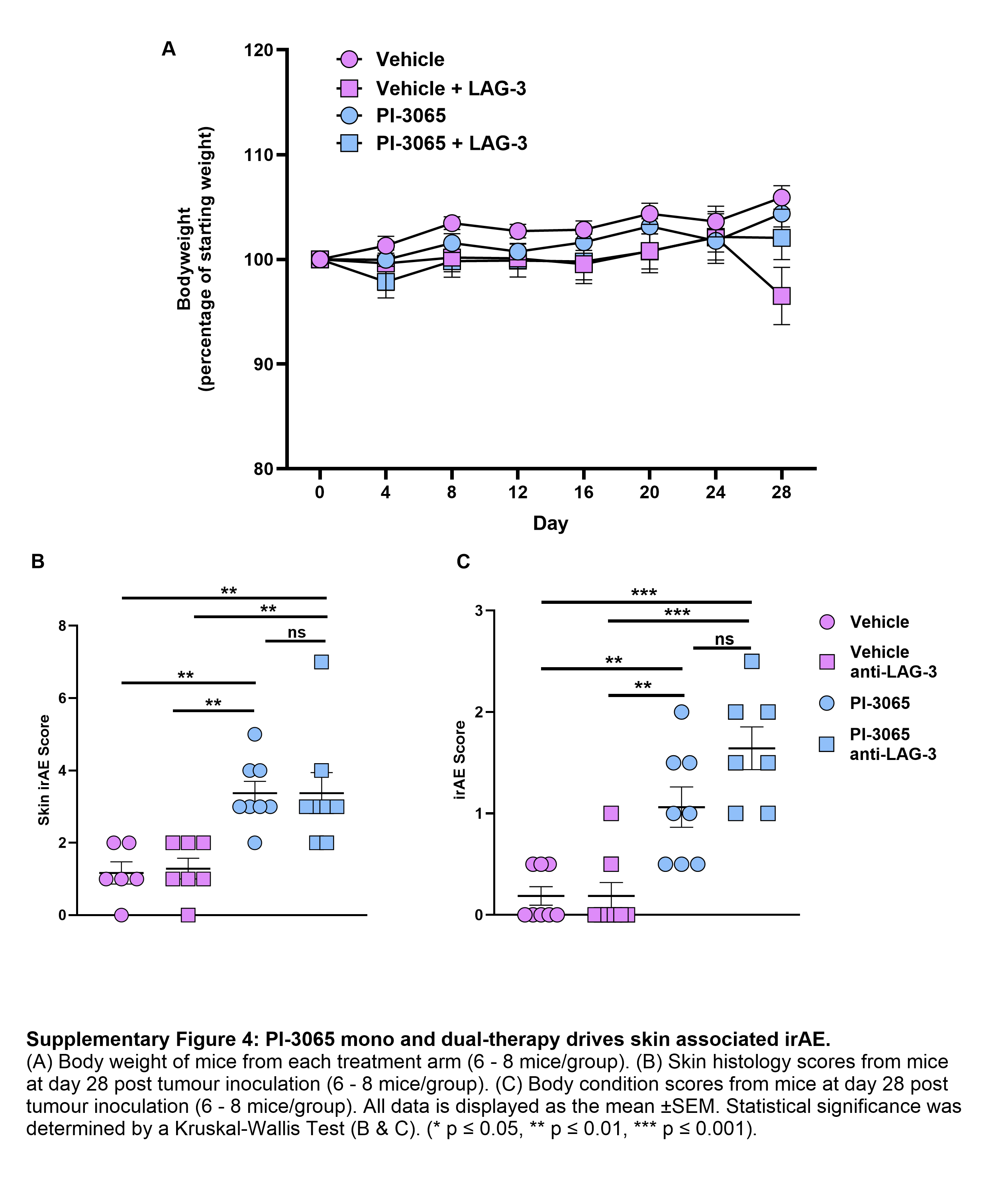

Supplement: online supplemental figure 4 [file jitc-14-2-s004.tif]

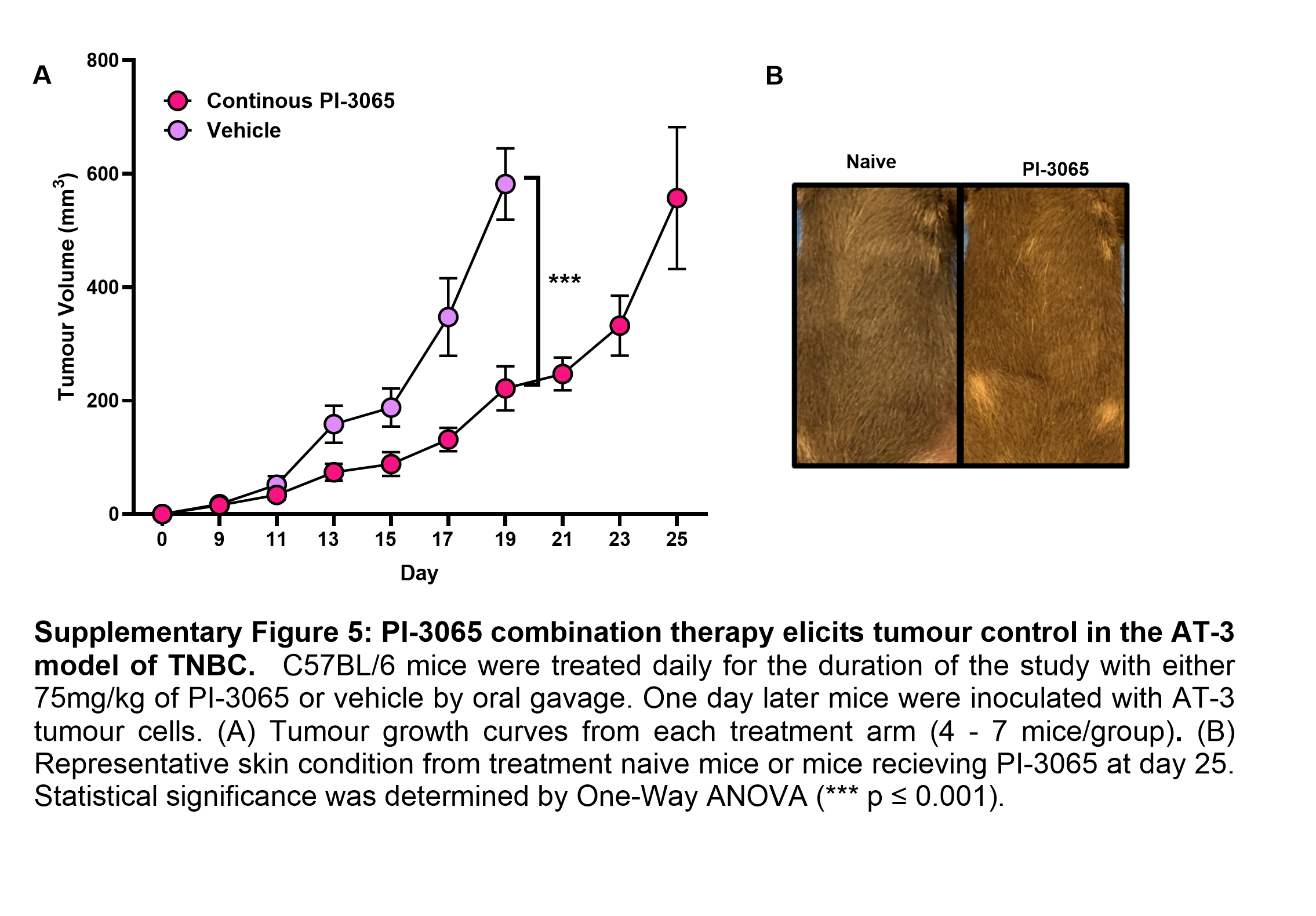

Supplement: online supplemental figure 5 [file jitc-14-2-s005.tif]

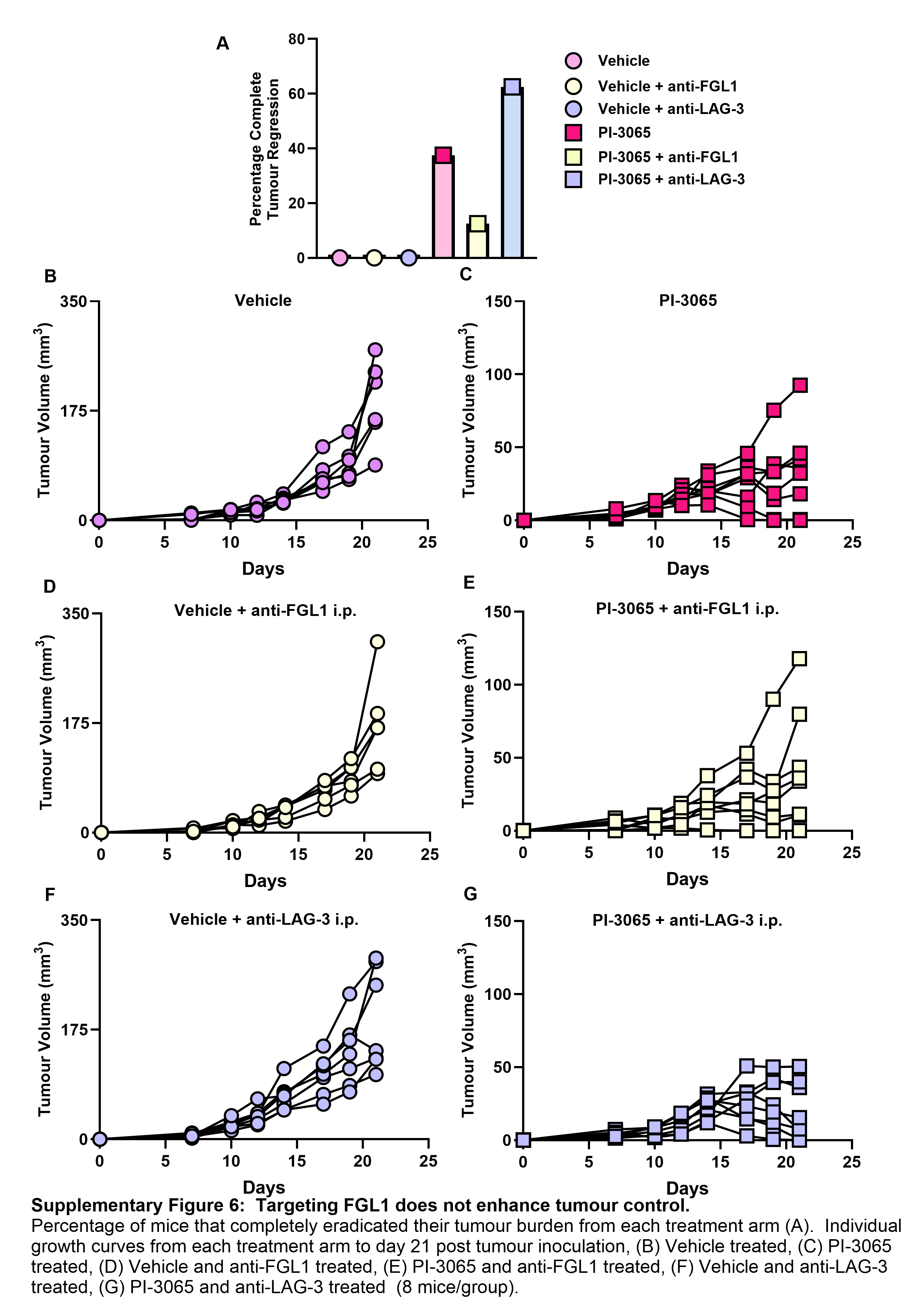

Supplement: online supplemental figure 6 [file jitc-14-2-s006.tif]

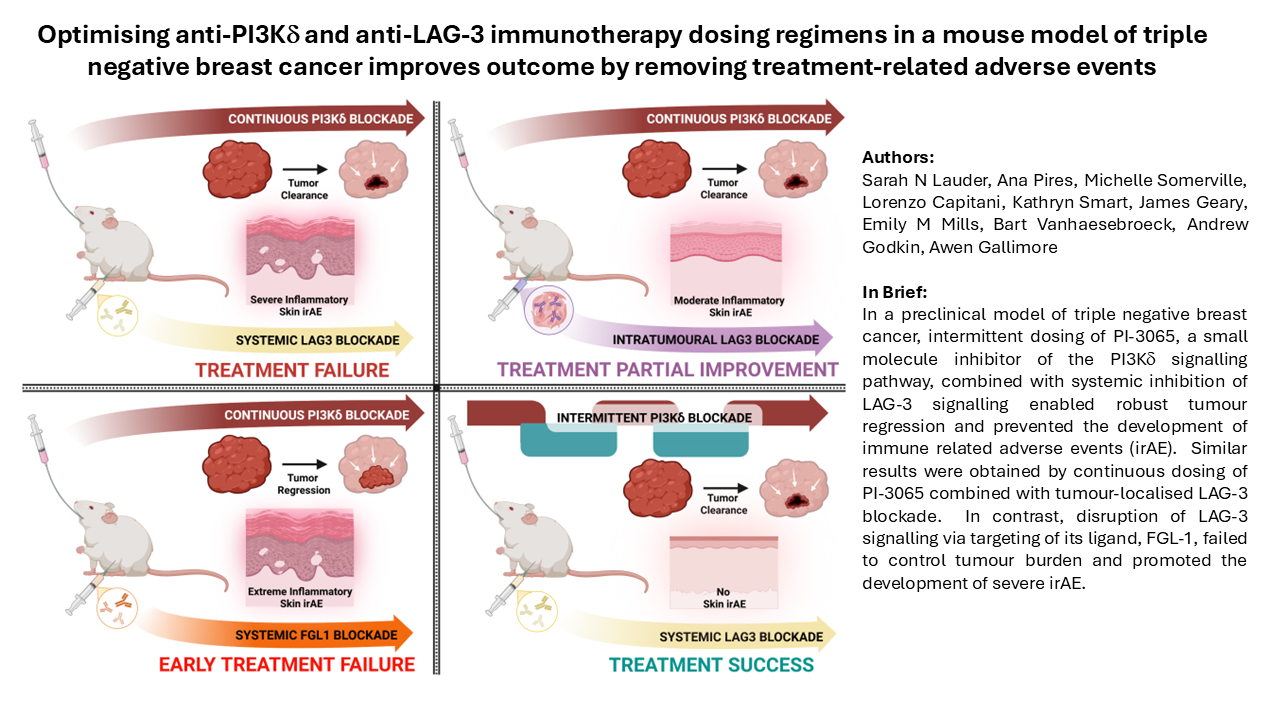

Supplement: online supplemental file 1 [file jitc-14-2-s008.tif]
