# Supplementary material for: Optimizing anti-PI3Kδ and anti-LAG-3 immunotherapy dosing regimens in a mouse model of triple-negative breast cancer improves outcome by removing treatment-related adverse events
Source: J Immunother Cancer. 2026 Feb 2;14(2):e012157. doi: 10.1136/jitc-2025-012157 (PMC12878477; doi:10.1136/jitc-2025-012157)
Supplement: online supplemental table 1 [file jitc-14-2-s007.pdf]

Treatment Group	ET	ED	EI	DI	SI	Total
PI-3065 22	0.5	0	0	0	0	0.5
PI-3065 23	0.5	0	0	0	0	0.5
PI-3065 24	0.5	0	0	0	0	0.5
PI-3065 19	0.5	1	0	0	0	1.5
PI-3065 18	1	0.5	0.5	0	0	2
PI-3065 4	1	0	0	1	0	2
PI-3065 13	0.5	1	1	0	0	2.5
PI-3065 10	1	0.5	1	0	0	2.5
PI-3065 5	1	1	0	1	0	3
PI-3065 8	1	1	0	1	0	3
PI-3065 20	1	1	1	0	0	3
PI-3065 3	1	1	0	1	0	3
PI-3065 11	1	1	1	0	0	3
PI-3065 16	1	1	1	0	0	3
PI-3065 17	1	1	1	0	0	3
PI-3065 2	1	1	0	1	0	3
PI-3065 21	1.5	1	1	0	0	3.5
PI-3065 12	1.5	1	1	0	0	3.5
PI-3065 9	1.5	1	1	0	0	3.5
PI-3065 14	2	1	1	0	0	4
PI-3065 1	1	1	0	2	0	4
PI-3065 6	1	1	1	1	0	4
PI-3065 15	2	1	2	0	0	5
PI-3065 7	1	1	2	2	0	6
PI-3065 + LAG3 3	1	0	0	0	0	1
PI-3065 + LAG3 2	1	0	1	0	0	2
PI-3065 + LAG3 4	1	0	0	1	0	2
PI-3065 + LAG3 9	1	1	0.5	0	0	2.5
PI-3065 + LAG3 1	1	0	1	1	0	3
PI-3065 + LAG3 6	1	1	0	1	0	3
PI-3065 + LAG3 8	1	1	0	1	0	3
PI-3065 + LAG3 10	1	1	1	0	0	3
PI-3065 + LAG3 18	1	1	1	0	0	3
PI-3065 + LAG3 22	1.5	1	0.5	0	0	3
PI-3065 + LAG3 16	1	1	1.5	0	0	3.5
PI-3065 + LAG3 19	1.5	1	1	0	0	3.5
PI-3065 + LAG3 20	1.5	1	1	0	0	3.5
PI-3065 + LAG3 21	1.5	1	1	0	0	3.5
PI-3065 + LAG3 5	1	1	1	1	0	4
PI-3065 + LAG3 14	1.5	1	1.5	0	0	4
PI-3065 + LAG3 23	1.5	1	1	1	0	4.5
PI-3065 + LAG3 15	1	1	2	1	0	5
PI-3065 + LAG3 12	1	1	2	1	0.5	5.5
PI-3065 + LAG3 17	2	1	2	1	0	6
PI-3065 + LAG3 7	2	1	1	2	1	7
PI3065 + LAG3 11	3	1	2	2	0.5	8.5
PI-3065 + LAG3 13	2	1	3	1	1	8

Supplementary Table 1: Individual Histopathological Skin Scores

5μM skin sections stained with haematoxylin and eosin from continuous PI-3065 treated mice or continuous PI-3065 + anti-LAG-3 antibody (i.p.) treated mice were scored blinded for epidermal thickening (ET), epidermal damage (ED), epidermal infiltrate (EI), dermal infiltrate (DI) and subcutaneous infiltrate (SI) using the scoring criteria detailed in the methods. The combined histological score was determined for each sample. Animals with minimal skin inflammation and are comparable to the scores of the control groups; vehicle and vehicle + anti-LAG3 ab treated mice are highlighted in blue. Animals with skin irAE scores that are induced by PI-3065 mono or combination therapy but are comparable between the two treatment groups are highlighted in yellow. Animals experiencing skin irAE that are more extensive and include subcutaneous infiltration are highlighted in red. 23 – 24 mice per group were scored, combined from independent experiments (Figures 6D, Figure 7D and Supplementary Figure 4B).
